# Plasma p217+tau accurately discriminates Intermediate or Advanced Alzheimer's Disease biological PET stages

**DOI:** 10.1002/alz.090530

**Published:** 2025-01-09

**Authors:** Azadeh Feizpour, Vincent Dore, Natasha Krishnadas, Pierrick Bourgeat, James D. Doecke, Ziad S. Saad, Gallen Triana‐Baltzer, Simon M. Laws, Rosita Shishegar, Kun Huang, Christopher J Fowler, Larry Ward, Colin L Masters, Jurgen Fripp, Hartmuth C. Kolb, Victor L Villemagne, Christopher C. Rowe

**Affiliations:** ^1^ Department of Molecular Imaging & Therapy, Austin Health, Melbourne, VIC Australia; ^2^ The Florey Institute of Neuroscience and Mental Health, Melbourne, VIC Australia; ^3^ The Australian e‐Health Research Centre, Commonwealth Scientific and Industrial Research Organisation, Melbourne, VIC Australia; ^4^ Department of Molecular Imaging & Therapy, Austin Health, Heidelberg, VIC Australia; ^5^ The Australian e‐Health Research Centre, Commonwealth Scientific and Industrial Research Organisation, Brisbane, QLD Australia; ^6^ The Australian e‐Health Research Centre, CSIRO, Brisbane, QLD Australia; ^7^ Johnson & Johnson Innovative Medicine, San Diego, CA USA; ^8^ Janssen Research & Development, LLC, La Jolla, CA USA; ^9^ Collaborative Genomics and Translation Group, School of Medical and Health Sciences, Edith Cowan University, Joondalup, Western Australia Australia; ^10^ Curtin Medical School, Curtin University, Bentley, Western Australia Australia; ^11^ Centre for Precision Health, Edith Cowan University, Joondalup, Western Australia Australia; ^12^ The Australian e‐Health Research Centre, CSIRO, Parkville, VIC Australia; ^13^ Austin Health, Melbourne, VIC Australia; ^14^ The Florey Institute of Neuroscience and Mental Health, Parkville, VIC Australia; ^15^ University of Pittsburgh, Pittsburgh, PA USA

## Abstract

**Background:**

Plasma phospho‐tau biomarkers, such as p217+tau, excel at identifying Alzheimer’s Disease (AD) neuropathology. However, questions remain regarding their capacity to inform AD biological PET stages at group level and maintain the same precision at individual patient level.

**Method:**

Participants included 248 cognitively unimpaired (CU) and 227 cognitively impaired (CI) individuals, with Janssen plasma p217+tau Simoa® assay, ^18^F‐NAV4694 Aβ PET (A) and ^18^F‐MK6240 tau PET (T) data. Biological PET stages were defined based on the draft NIA‐AA Revised Criteria (July 2023): Initial (A+T‐), Early (A+T_MTL_+), Intermediate (A+T_MOD_+), and Advanced (A+T_HIGH_+). We used thresholds for A+ of 25 Centiloid and for T_HIGH_ of 80 Centaur (2.68 SUVR_temporo‐parietal_). Adding an A‐T‐ stage for comparison, we assessed the performance of p217+tau in discriminating between these stages at the group level using Receiver Operating Characteristic (ROC) analysis and at the individual level using logistic regression.

**Result:**

Plasma p217+tau concentrations increased across the stages, with significant differences between them, except for the Initial and Early stages. Screening for Advanced (vs. lower stages), combined Intermediate/Advanced (vs. lower stages), or all stages (vs. A‐T‐), p217+tau showed good group‐level discriminations (AUC 0.91, 0.92 and 0.92; CI only: AUC 0.83, 0.89, 0.93, respectively). At the individual level, the likelihood of PET stage vs. p217+tau level showed good discrimination of A‐T‐ vs any A+ stage and of combined Intermediate/Advanced disease stage vs lower stages in the CI.

**Conclusion:**

In addition to accurately screening for A+ individuals, plasma p217+tau shows promise for separating persons with either Intermediate or Advanced stage AD from those at a lower stage, providing prognostic information and informing better selection for trials and disease modifying therapies.

